# Untargeted metabolomic on urine samples after α-lipoic acid and/or eicosapentaenoic acid supplementation in healthy overweight/obese women

**DOI:** 10.1186/s12944-018-0750-4

**Published:** 2018-05-09

**Authors:** Ana Romo-Hualde, Ana E. Huerta, Carlos J. González-Navarro, Omar Ramos-López, María J. Moreno-Aliaga, J. Alfredo Martínez

**Affiliations:** 1 Centre for Nutrition Research, University of Navarra, Pamplona, Spain; 2 Department of Nutrition, Food Science, and Physiology, University of Navarra, Pamplona, Spain; 3 Spanish Biomedical Research Centre in Physiopathology of Obesity and Nutrition (CIBERobn), Institute of Health Carlos III (ISCIII), Madrid, Spain; 4 Navarra Institute for Health Research (IDISNA), Pamplona, Spain; 5 Madrid Institute of Advanced Studies (IMDEA Food), Madrid, Spain

**Keywords:** α-lipoic acid, Eicosapentaenoic acid, Obesity, Overweight, Metabolomic

## Abstract

**Background:**

Eicosapentaenoic acid (EPA) and α-lipoic acid (α-LA) have been investigated for their beneficial effects on obesity and cardiovascular risk factors. In the current research, the goal was to evaluate metabolomic changes following the dietary supplementation of these two lipids, alone or combined in healthy overweight/obese sedentary women following an energy-restricted diet. For this purpose, an untargeted metabolomics approach was conducted on urine samples using liquid chromatography coupled with time of flight mass spectrometry (HPLC-TOF-MS).

**Methods:**

This is a short-term double blind placebo-controlled study with a parallel nutritional design that lasted 10 weeks. Participants were assigned to one of the 4 experimental groups [Control, EPA (1.3 g/d), α-LA (0.3 g/d) and EPA+α-LA (1.3 g/d + 0.3 g/d)]. All intervention groups followed an energy-restricted diet of 30% less than total energy expenditure. Clinically relevant biochemical measurements were analyzed. Urine samples (24 h) were collected at baseline and after 10 weeks. Untargeted metabolomic analysis on urine samples was carried out, and principal component analysis (PCA) and partial least squares-discriminant analysis (PLS-DA) were performed for the pattern recognition and characteristic metabolites identification.

**Results:**

Urine samples were scattered in the PCA scores plots in response to the supplementation with α-LA. Totally, 28 putative discriminant metabolites in positive ionization, and 6 in negative ionization were identified among groups clearly differentiated according to the α-LA administration. Remarkably is the presence of an ascorbate intermediate metabolite (one of the isomers of trihydroxy-dioxohexanoate, or dihydroxy–oxohexanedionate) in the groups supplemented with α-LA. This fact might be associated with antioxidant properties of both α-LA and ascorbic acid. Correlations between phenotypical parameters and putative metabolites of provided additional information on whether there is a direct or inverse relationship between them. Especially interesting are the negative correlation between ascorbate intermediate metabolite and asymmetric dimethylarginine (ADMA) and the positive one between superoxide dismutase (SOD) and α-LA supplementation.

**Conclusions:**

This metabolomic approach supports that the beneficial effects of α-LA administration on body weight reduction may be partly explained by the antioxidant properties of this organosulfur carboxylic acid mediated by isomers of trihydroxy-dioxohexanoate, or dihydroxy–oxohexanedionate.

**Trial registration:**

Clinicaltrials.gov NCT01138774.

**Electronic supplementary material:**

The online version of this article (10.1186/s12944-018-0750-4) contains supplementary material, which is available to authorized users.

## Background

Metabolomics is an – omics technology that focuses on analyzing the wide variety of low molecular weight metabolites occurring in biological samples (urine, blood, tissues, etc.) [1]. Targeted metabolomic is a powerful tool to quantify of known metabolites with similar chemical structures (e.g. amino acids, acylcarnitines, organic acids, etc.) [2], while untargeted metabolomic involves the use of nuclear magnetic resonance (NMR) or mass spectrometry (MS) for the simultaneous measurement of as many known and unknown metabolites as possible in a biological specimen [3]. This latter approach is generally used to compare biological samples or clinical states and to report on differences between these situations in order to assess the complete set of metabolites – metabolome [4]. Two factors that influence the metabolome have been described: the endogenous metabolome, which involves all intrinsic metabolites related to the primary and intermediary metabolism, and the exogenous metabolome, which refers to all metabolites arising from extrinsic factors such as diet (i.e., food metabolome), microbiota, physical activity, stress, or drugs [5]. Metabolomics promises to be an important instrument in interpreting the complex relationships between factors contributing to different diseases: obesity, diabetes, cancer, etc. [6]. In this context, preliminary results on metabolomic biomarkers related to the different dietary patterns could help to better understand the inter-individual differences in cardiovascular risk and nutritional responses for further applications in precision nutrition [7].

Diverse fatty acids have been widely investigated in a number of clinical trials for their beneficial effects on obesity [8] and cardiovascular risk factors [9]. Thus, α-lipoic acid (α-LA) is a naturally occurring carboxylic acid with antioxidant properties, which contains sulfur and eight carbons, being a co-factor in different mitochondrial enzymes [10]. The human body can synthesize small amounts of α-LA through lipoic acid synthase [11]. Some studies in rodents have described anti-obesity properties for α-LA supplementation, and also beneficial effects on hepatic steatosis, which could be mediated by its ability to restore the oxidative balance by increasing antioxidant defenses [12, 13]. It has also been shown that α-LA down regulates lipogenic enzymes, inhibiting lipogenesis and reducing triglyceride accumulation (through the activation of AMP-activated protein kinase (AMPK) signaling pathway) in human subcutaneous adipocytes from overweight/obese subjects [14]. Interestingly, several meta-analyses on clinical trials carried out to study the effect of α-LA supplementation on obesity and overweight [15, 16] revealed that supplementation with α-LA slightly, but significantly decreased body weight and body mass index (BMI) [15, 16]. Further research is warranted to examine the effect of different doses and the long-term benefits of LA on weight management [16].

Other fatty acid such as eicosapentaenoic acid (EPA), which is one of the principal omega-3 polyunsaturated fatty acids (n-3 PUFA) from marine origin, is associated with anti-inflammatory properties [17]. In this context, an intervention trial has shown that EPA modulates inflammation-related genes in adipose tissue [18]; moreover, EPA promotes changes in the adipose tissue extracellular matrix remodeling genes besides an increment of chemotactic factors and macrophages associated with wound repair [19]. Different metabolomic studies have been carried out on EPA and n-3 PUFA [9, 20]. Thus, a lipidomic study contributed to the general knowledge of EPA on the progress of metabolic syndrome (MetS), inflammation and oxidative stress [20]. A human trial showed indirect associations with lipid molecular species and clinical variables of interest in the evaluation of the MetS after a diet high in n-3 PUFA and polyphenols [9]. Therefore, the aim of the present study was to assess the effect of dietary supplementation with α-LA and EPA, separately or in combination during a hypocaloric diet, on urinary metabolome, in order to evaluate the presence of metabolomic changes between the different groups of the intervention.

## Methods

### Participants and study design

The current analyses were conducted within the framework of the OBEPALIP project [21], which is a double blind randomized placebo-controlled intervention with a parallel nutritional design, where a group of 70 healthy overweight/obese sedentary females (37.3 ± 7.6 years old, and 31.6 ± 3.1 BMI) was chosen. All volunteers followed an energy-restricted diet of 30% E adjusted with the individual’s energy expenditure assessed by indirect calorimetry [21] during 10 weeks. Macronutrient distribution was recommended according to the American Heart Association (AHA) nutritional guidelines [22]. At the initial baseline visit, the experimental prescription was personally taught by a trained dietitian and all participants were instructed about maintaining the habitual physical activity style over the trial. Furthermore, in this first visit, participants were allocated to one of the four intervention groups: 1) Control group (*n* = 19): 3 placebo-I capsules (containing sunflower oil) and 3 placebo-II capsules (containing the same excipients as the LA capsules); 2) EPA group (*n* = 15): 1300 mg/d of EPA distributed in 3 capsules of EPA80 (provided by Solutex®, Madrid, Spain), supplying 433.3 mg of EPA and 13.8 mg of DHA as ethyl-ester; and 3 placebo-II capsules; 3) α-LA group (*n* = 16): 300 mg/d of α-LA from 3 capsules supplying 100 mg of α-LA (Nature’s Bounty®, NY, USA), and 3 placebo-I capsules; and 4) EPA + α-LA (*n* = 15): 1300 mg/d of EPA (distributed in 3 capsules of EPA80) and 300 mg/d of α-LA (from 3 capsules containing 100 mg of α-LA), respectively [18, 23].

At the baseline and at the endpoint, the women, after about 10–12 h fasting, visited the Metabolic Unit of the University of Navarra to be interviewed by the physician, the dietitian and the nurse. Anthropometric measurements were carried out according with standardized routine protocols, as detailed elsewhere [18, 23]. The inclusion and exclusion criteria have been previously described [21].

The study was approved by the Research Committee of the University of Navarra **No. 007/2009** and recorded at clinicaltrials.gov as **NCT01138774**. All undertakers signed the informed consent before being recruited in the assay. The intervention was conducted in accordance with the latest Helsinki Declaration guidelines.

### Biochemical measurement in blood and urine

Blood specimens from overnight fasted subjects were drained on weeks 0 and 10 into Serum Clot Activator tubes (4 mL Vacuette®) and into tubes with tripotassium EDTA (4 mL Vacuette®). Plasma samples were extracted from EDTA tubes after centrifugation at 1500 g during 15 min at 4 °C. All samples were adequately stored at − 80 °C for posterior appropriate analyses.

Serum concentrations of glucose, total cholesterol, HDL-cholesterol, triglycerides, free fatty acids (FFA) and β-hydroxybutyrate were routinely assessed by using the Pentra C200 (HORIBA medical, Madrid, Spain) auto-analyzer. The values of LDL-cholesterol were calculated using the Friedewald equation. Also, plasma concentrations of asymmetric dimethylarginine (ADMA) and insulin were measured according with the manufacturer’s instructions for available commercial ELISA kits provided by DLD Diagnostika GMBH (Hamburg, Germany) and Mercodia (Uppsala, Sweden), respectively [18]. The homeostasis model assessment (HOMA-IR) was defined as fasting serum insulin (mU/L) x fasting plasma glucose (mmol/L)/22.5 [24]. Superoxide dismutase (SOD) activity was measured with a kit according to manufacturer instructions (Assay Designs, PA, USA) as described elsewhere [21]. ADMA was assessed in the samples as marker of metabolic syndrome manifestations an oxidative status [25], and SOD was measured as biological marker of oxidative stress [26], as positively related with the α-LA antioxidant properties.

Complete 24 h urine samples were picked up the day prior to the start and the day before the endpoint of the study. Urine specimens were collected in a urine container and chilled at 4 °C. As designed, the urine samples were stored in vials of 1 mL at − 80 °C until analysis.

### Sample preparation and HPLC-TOF-MS analysis

All used solvents were of liquid chromatography-mass spectrometry (LC-MS) grade and purchased from Scharlau (Scharlab, Sentmenat, Spain). Analytical water (18.2 MΩ) was provided from an Ultramatic system from Wasserlab (Barbatáin, Navarra, Spain). Other standards were of analytical or higher grade, and were supplied by Sigma Aldrich (Sigma-Aldrich Chemie Gmbh, Steinheim, Germany).

The analytical procedures have been thoroughly described elsewhere [27]. In brief, urine samples were warmed up, centrifuged, diluted with 100 μL of Milli-Q water and vigorously vortexed. The solution was finally transferred to a vial for subsequent analyses. High Performance Liquid Chromatographic (Agilent Technologies 1200, CA, USA) coupled with Mass Spectrometry (Agilent Technologies 6220 Accurate-Mass TOF LC-MS, CA, USA) was available for the analysis. MS operated in positive electrospray ionization (ESI+) or negative electrospray ionization (ESI-) mode. The HPLC-TOF-MS system was controlled by MassHunter Workstation 06.00 software (Agilent Technologies, Barcelona, Spain). The used column was a Zorbax SB-C18 (15 cm × 0.46 cm; 5 μm) with a SB-C18 precolumn (Agilent Technologies, Barcelona, Spain) at 40 °C. The mobile phase involved water with formic acid 0.1% (A) and acetonitrile with formic acid 0.1% (B). The gradient for elution, 1–20% B, 0–4 min, 20–95% B 4–6 min, 95% B 9–7.5 min, 95–1% B 7.5–8 min, 1% B 8–14 min. Afterwards, the column was re-equilibrated for 5 min at 1% B. The flow rate was 0.6 mL min^− 1^ and the injection volume was 15 μL. ESI conditions were: gas temperature, 350 °C; drying gas, 10 L min^− 1^; nebulizer, 45 psig; capillary voltage, 3500 V; fragmentor, 175 V; and skimmer, 65 V. The equipment was set out to work over the m/z range 100–2000 with an acquisition rate of 1.03 spectra s^− 1^.

To assess the quality in this metabolomic approach, a previously reported procedure with some minor modifications was applied [28, 29]. Two kinds of sample quality control (QCs) were implemented: i) standard mixture solution implemented of cytosine, L-carnitine hydrochloride, betaine, leucine, deoxyadenosine and deoxyguanosine at a concentration of 1 mg/L. ii) pooled urine was prepared by mixing equal volumes from each of the 130 samples. These samples were injected 5 times at the beginning of the run to ensure system equilibration, and then every 5 samples to further monitor stability of the analysis. Finally, samples were randomized to reduce the systematic error associated with measurements variability. Urine specimens were sequentially analyzed in sets of 15 samples/day.

### Data processing and metabolite identification

LC-MS data were analyzed with the XCMS Online software (https://xcmsonline.scripps.edu) to identify and line up features [30–34]. The alignment applied a 0.2 min retention time and a 0.002 Da mass tolerance window.

A pilot trial was performed to characterize metabolites by means of the METLIN (https://metlin.scripps.edu/index.php) within a mass precision below 5 mDa, the scientific literature and the metabolic pathways described in Kyoto Encyclopaedia of Genes and Genomes (KEGG) database (http://www.genome.jp/kegg/), Human Metabolome Database (HMDB) (http://www.hmdb.ca/) and Lipidmaps (http://www.lipidmaps.org/). In those cases in which the METLIN search offered several metabolites, the use of commercial patterns allowed us to discard some of the resulting options, allowing a more accurate approximation to the putative metabolite.

### Statistical analysis

Statistical analyses were carried out with the Stata Statistical Software (Release 12. College Station, StataCorp LLC, TX, USA). For all performed tests, the statistical significance (two-sided) was set at *p* < 0.05. The Shapiro-Wilk analysis was used to check the sample normality. Anthropometric and biochemical parameters at baseline were compared between groups by a one-way analysis of variance (ANOVA) or Kruskal-Wallis tests, as statistically appropriate. Moreover, the percentage of change, defined as [(endpoint-baseline) / baseline] × 100, was compared by two-way ANOVA and adjusted by the respective value at baseline when proper. Moreover when a statistically significant interaction was found (EPA x LA), a contrast analysis was applied to identify which conditions were different from each other.

Metabolomic profile examination encompassed diverse multivariate data analysis procedures such as principal components analysis (PCA) and partial least squares-discriminant analysis (PLS-DA). Such analyses were implemented using MetaboAnalyst 3.0 software (http://www.metaboanalyst.ca/). Before carrying out PCA and PLS-DA analyses, the peak intensity was controlled by a logarithmic transformation, and monitored by Pareto scaling. To research the more relevant metabolites in the PLS-DA model, variable importance in projection (VIP) scores were estimated. Metabolites with VIP score value greater than 4.0 were chosen for proof of identity. In addition, Spearman’s correlations were performed between phenotypical parameters and putative metabolites.

## Results

### Subjects

The principal characteristics of participants and biochemical parameters at baseline and changes after the 10 week intervention concerning the 4 experimental arms following hypocaloric diets and α-LA/EPA administration are reported (Table 1). The four experimental groups were apparently homogeneous at the baseline, where no statistical differences were found at the beginning of the trial in any of the assessed variables. BMI, fat mass and HOMA-IR reductions were significantly higher (*p* < 0.05) in those groups supplemented with α-LA while it was observed a significant interaction (p < 0.05) between treatments in the percentage of change of ADMA levels.

**Table 1:** Biochemical and anthropometric characteristics of volunteers at baseline and percent of change

Parameters	Control (*n* = 19)	EPA (*n* = 15)	α-LA (*n* = 16)	EPA+ α-LA (*n* = 15)	Two-way ANOVA		
					EPA	α-LA	EPA x α-LA
*Age (years)* ^*a*^	39.0 ± 8.0	37.2 ± 8.1	39.3 ± 6.6	38.1 ± 7.0	ns	ns	ns
*Body Mass Index*							
Baseline^b^	33.3 ± 6.1	32.9 ± 3.1	32.5 ± 4.2	33.2 ± 3.7			
Endpoint^c^	31.1 ± 6.0	30.9 ± 3.3	29.8 ± 4.0	30.6 ± 3.8	ns	ns	ns
Change (%)^c^	−6.6 ± 2.9	−6.1 ± 2.7	−8.2 ± 3.6	−7.9 ± 3.5	ns	0.036	ns
*Fat mass*							
Baseline (kg)^a^	36.8 ± 10.7	37.8 ± 7.2	35.8 ± 9.6	36.7 ± 9.3			
Endpoint (kg)^c^	32.5 ± 10.6	33.8 ± 7.8	30.1 ± 8.6	31.1 ± 8.4	ns	ns	ns
Change (%)^c^	−12.2 ± 5.8	−11.2 ± 7.0	−15.8 ± 7.8	− 15.4 ± 6.5	ns	0.025	ns
*Waist circumference*							
Baseline (cm)^a^	100.8 ± 14.9	101.9 ± 7.8	96.6 ± 9.2	98.5 ± 9.0			
Endpoint (cm)^c^	95.1 ± 15.3	95.0 ± 8.2	90.7 ± 8.9	91.8 ± 8.9	ns	ns	ns
Change (%)^c^	−5.8 ± 2.4	−6.8 ± 3.4	−6.0 ± 3.4	−6.7 ± 2.9	ns	ns	ns
*HOMA-IR*							
Baseline^a^	1.9 ± 1.9	1.5 ± 1.1	1.5 ± 0.7	2.0 ± 1.1			
Endpoint^c,d^	1.2 (0.2)	1.4 (0.2)	1.1 (0.2)	1.1 (0.2)	ns	ns	ns
Change (%)^c,d^	−21.1 (9.6)	3.1 (10.8)	−29.0 (10.4)	−37.1 (10.8)	ns	0.024	ns
*LDL-cholesterol*							
Baseline (mg/dL)^b^	125.6 ± 32.0	119.6 ± 34.1	122.9 ± 25.7	129.8 ± 35.8			
Endpoint (mg/dL)^c^	119.4 ± 31.9	102.0 ± 28.1	107.4 ± 19.7	113.7 ± 34.1	ns	ns	ns
Change (%)^c^	−4.5 ± 10.7	−13.8 ± 14.2	−10.7 ± 18.3	−11.9 ± 15.0	ns	ns	ns
*HDL-cholesterol*							
Baseline (mg/dL)^b^	50.4 ± 11.1	50.4 ± 10.3	49.6 ± 9.9	49.0 ± 13.6			
Endpoint (mg/dL)^c^	47.2 ± 9.8	44.7 ± 11.9	42.8 ± 10.5	44.8 ± 10.6	ns	ns	ns
Change (%)^c^	−5.4 ± 11.8	−11.1 ± 13.8	−13.8 ± 9.1	−6.7 ± 12.9	ns	ns	ns
*FFA*							
Baseline (mmol/L)^a^	0.51 ± 0.20	0.51 ± 0.10	0.52 ± 0.23	0.58 ± 0.18			
Endpoint (mmol/L)^c^	0.52 ± 0.19	0.57 ± 0.15	0.49 ± 0.14	0.63 ± 0.18	ns	ns	ns
Change (%)^c^	12.8 ± 45.2	13.4 ± 31.0	1.9 ± 0.17	31.6 ± 30.5	ns	ns	ns
*Triglycerides*							
Baseline (mg/dL)^a^	89.2 ± 41.6	84.4 ± 33.5	95.9 ± 50.7	91.3 ± 44.8			
Endpoint (mg/dL)^c,d^	77.4 (5.6)	77.2 (6.5)	70.0 (6.3)	69.7 (6.3)	ns	ns	ns
Change (%)^c,d^	−10.3 (6.4)	−7.4 (7.5)	−10.7 (7.2)	−17.8 (7.2)	ns	ns	ns
*β-hydroxybutyrate*							
Baseline (mmol/L)^a^	0.36 ± 0.26	0.28 ± 0.23	0.39 ± 0.23	0.32 ± 0.16			
Endpoint (mmol/L)^c^	0.28 (0.07)	0.45 (0.08)	0.33 (0.08)	0.42 (0.08)	ns	ns	ns
Change (%)^c,d^	20.9 (47.1)	70.5 (51.9)	32.1 (50.2)	123.8 (51.6)	ns	ns	ns
*ADMA*							
Baseline (μmol/L*)*^a^	0.58 ± 0.23	0.80 ± 0.45	0.57 ± 0.21	0.59 ± 0.23			
Endpoint (μmol/L)^c,d^	0.46 (0.05)	0.63 (0.06)	0.41 (0.06)	0.35 (0.06)	ns	0.007	ns
Change (%)^c,d^	−25.7 (10.8)	15.2 (12.5)	−33.7 (11.7)	−42.6 (12.1)*	–	–	0.04
*SOD*							
Baseline (U/mg)^a^	305.2 ± 275.3	195.3 ± 91.3	366.0 ± 510.8	245.1 ± 195.9			
Endpoint (U/mg)^c,d^	247.8 (38.1)	235.6 (44.7)	221.6 (41.9)	244.6 (44.4)	ns	ns	ns
Change (%)^c,d^	2.1 (6.5)	10.1 (7.6)	−1.7 (7.1)	0.5 (7.6)	ns	ns	ns

*Abbreviations*: *HOMA-IR* homeostasis model assessment, *FFA* free fatty acids, *ADMA* asymmetric dimethylarginine, *SO*D superoxide dismutase

Data are represented as mean ± SD if unadjusted or as mean (SE) if adjusted. At baseline no differences were observed between groups.^a^Kwallis; ^b^One-way ANOVA; ^c^Two-way ANOVA; ^d^Mean (SE) and adjusted by the respective value at baseline; *Different from EPA group

### Urinary metabolomic profile

HPLC-TOF-MS method allowed the detection of 4.752 features in the ESI+ mode and 4.713 features in the ESI- mode (data not shown). Furthermore, a univariate statistical analysis performed to select those variables demonstrating significant differences (p < 0.05) among groups, found 711 features in ESI+ mode and 829 features in ESI- mode.

**Fig. 1:**
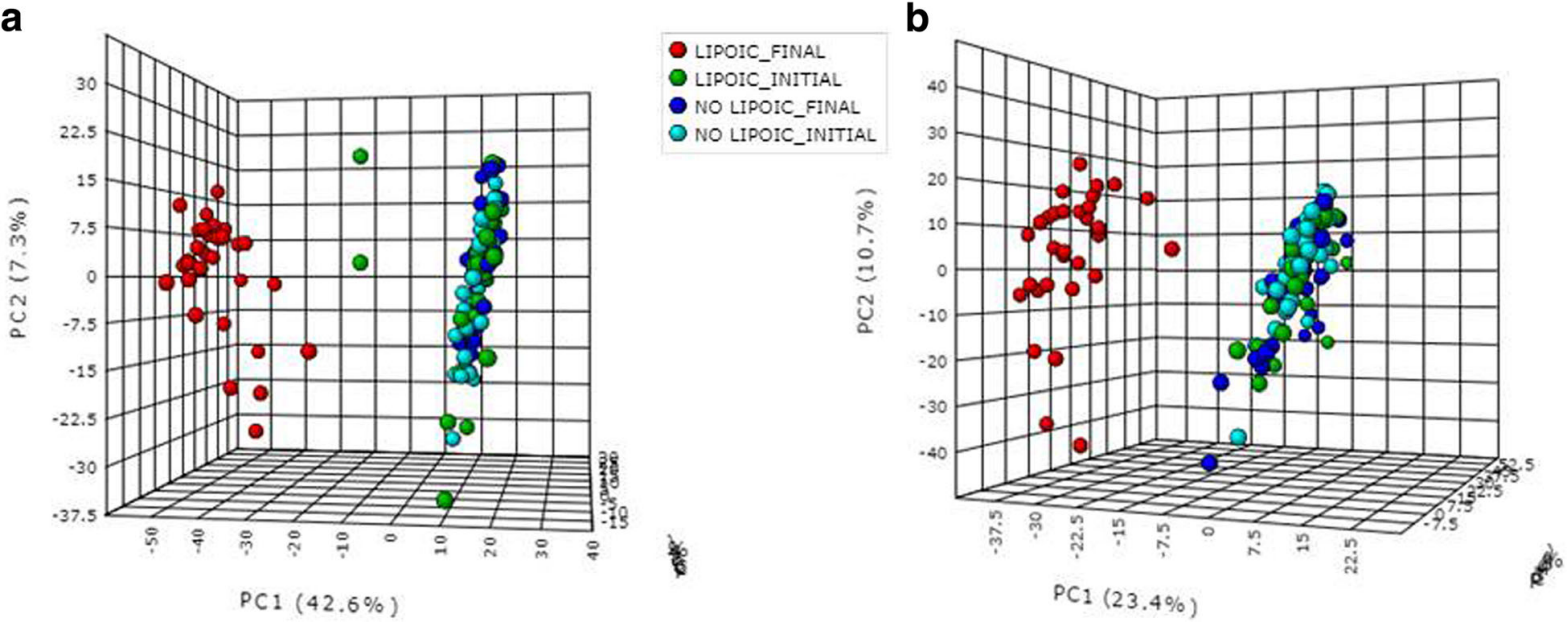
Principal component analysis (PCA) of untargeted metabolomics analysis of urine samples, including LIPOIC FINAL, LIPOIC INITIAL, NO LIPOIC FINAL and NO LIPOIC INITIAL groups. **a** PCA in positive ionization mode (ESI+). **b** PCA in negative ionization mode (ESI-)

A PCA approach was used to transform the original variables into a small number of new orthogonal variables built from linear combinations explaining most of the measured data variance [35], allowing the clustering of samples from groups [36]. Initially, PCA was able to discriminate the α-LA and the α-LA + EPA groups from the control and the EPA groups in both, positive and negative ionization mode, but was not able to discriminate among the four groups at endpoint (Additional file 1: Figure S1). Therefore, the α-LA and α-LA + EPA groups in a lipoic group (LIP) were gathered, while on the other hand the control and EPA groups in a non lipoic group (NO LIP) were also merged. Using these new groups, PCA showed a clear discrimination, both in the positive and negative ionization mode, between the LIP group at endpoint and the other three ones (LIP group at baseline, NO LIP group at endpoint and LIP group at baseline [Fig. 1]). Thus, it seems that the discriminant metabolites among groups were related to the metabolism of α-LA, and not influenced either by the energy restriction or, by the EPA treatment.

In order to identify the metabolites responsible for the discrimination among the metabolomic profiles, the VIP score was used to select those with the most significant contribution in a PLS-DA model. VIP scores are a weighted sum of PLS weights for each variable and measure the contribution of each predictor variable to the model. The compounds exhibiting the higher VIP score are the more influent variables. In this work, VIP score > 4 were used as a criterion for feature selection that was met by 28 features in ESI+ mode, and 6 features in ESI-mode thus selected for further identification.

### Identification of putative metabolites

Metabolites were putatively identified based on the information obtained from several databases, specifically the METLIN database was consulted (https://metlin.scripps.edu/index.php). Putative metabolites were detected when the mass difference between the theoretical m/z and detected m/z did not exceed 5 mDa. Some possible identity assignations were discarded by using commercial standards (citric acid, d-saccharic acid-1,4,-lactone). Table 2 (ESI+) and Table 3 (ESI-) contain information concerning the mean intensity of each metabolite at LIP and NO LIP baseline as well as LIP and NO LIP endpoint, retention time, detected mass, putative metabolite identification, assignation, mass difference and VIP score. Interestingly, all the putative metabolites with the highest VIP score presented an up-regulation in the LIP group at the endpoint. This fact supported the hypothesis raised above that the discriminant metabolites among the groups are associated with the intake of α-LA. Several putative metabolites were associated with dipeptides (tryptophan proline, or prolyl cysteine), CHO metabolites and lipid species. One of the identified putative metabolite, isomers of trihydroxy-dioxohexanoate (2,3-diketo-L-gulonate; 2,5-Didehydro-D-gluconate) or dihydroxy-oxohexanedioate (5-dehydro-4-deoxy-D-glucarate; 2-dehydro-3-deoxy-D-glucarate), was considered especially interesting, as it was increased at the LIP endpoint group compared to the other groups.

**Table 2:** Putative metabolites in ESI+

	NO LIP INI	LIP INI	NO LIP FIN	LIP FIN	VIP score	RT (min)	Detected mass (m/z)	Putative metabolites	Assignation	Mass difference (mDa)
1	10.01	10.03	15.48	19.31	6.833	9.05	402.1331	N-Methyl-2,3,7,8-tetramethoxybenzophenanthridine-6(5H)-one	[M + Na]^+^	1.90
								Angoline	[M + Na]^+^	1.90
								Asn-OHPhe-OH	[M + H]^+^	3.54
2	10.76	12.26	15.28	20.32	6.785	6.13	241.0614	Prolyl-Cysteine	[M + Na]^+^	−0.30
3	nd	nd	13.98	18.87	6.751	9.07	367.0961	Sanaganone	[M + Na]^+^	2.00
								4′,6′-Dihydroxy-2′-methoxyacetophenone 6′-glucoside	[M + Na]^+^	−3.90
4	10.11	9.39	13.37	17.95	6.538	8.66	475.0791	Unknown		
5	12.14	12.62	15.35	18.96	6.367	9.04	385.1076	8-p-Coumaroyl-3,4-dihydro-5,7-dihydroxy-4-phenylcoumarin	[M + H-H_2_O]^+^	0.00
								Asp-OHPhe-OH	[M + H-H_2_O]^+^	4.00
								Val-His-OH	[M + Na]^+^	−4.30
6	9.78	9.64	15.45	19.06	6.348	8.24	686.1217	Unknown		
7	9.61	9.87	12.50	16.28	6.212	8.69	518.1319	Unknown		
8	6.18	10.02	12.87	17.19	6.208	9.40	475.0599	S-antazirine	[M + H-H_2_O]^+^	0.90
9	14.05	13.87	16.81	20.43	6.182	8.23	165.0072	Chloro-methylphenol	[M + Na]^+^	−0.60
10	8.84	10.48	14.56	19.00	6.164	8.19	209.0358	Ethyl-2-amino-4-methyl-Thiazole-5-Carboxylate	[M + Na]^+^	0.30
								2-Chloro-1,3-dimethoxy-5-methylbenzene	[M + Na]^+^	1.80
11	13.02	12.90	15.26	18.79	6.139	8.73	336.1393	Aspartylglycosamine	[M + H]^+^	−0.80
								Methionine Tryptophan dipeptide	[M + H]^+^	1.70
12	4.29	8.58	11.92	16.21	6.091	9.40	477.0582	Cyanidin-3-arabinoside	[M + Na]^+^	2.30
								1. 5-Amino-4-imidazole-N-succinocarboxamide ribonucleotide	[M + Na]^+^	−4.70
13	11.17	11.23	14.77	18.62	6.018	7.84	197.0354	Unknown		
14	9.78	9.79	15.86	19.93	5.939	8.69	193.0390	2,3-Diketo-L-gulonate (isomers of trihydroxy-dioxohexanoate)	[M + H]^+^	4.70
								2,5-Didehydro-D-gluconate (isomers of trihydroxy-dioxohexanoate)	[M + H]^+^	4.70
								5-dehydro-4-deoxy-D-glucarate (isomers of dihydroxy-oxohexanedioate)	[M + H]^+^	4.70
								2-dehydro-3-deoxy-D-glucarate (isomers of dihydroxy-oxohexanedioate)	[M + H]^+^	4.70
15	13.17	12.33	15.60	19.30	5.890	8.74	324.1363	Tryptophan Proline dipeptide	[M + Na]^+^	4.40
16	9.44	9.33	13.67	17.22	5.869	8.24	688.1193	Unknown		
17	10.47	12.50	11.67	15.63	5.738	8.26	404.1056	Asn-Tyr-OH	[M + H]^+^	−3.20
18	13.05	12.28	13.52	17.48	5.712	8.22	424.0814	Xanthommatin	[M + H]^+^	3.90
19	11.35	5.29	12.98	18.44	5.697	9.05	407.0891	7-Chloro-3,4′,5,6,8-pentamethoxyflavone	[M + H]^+^	−0.10
								2-[6-(4′-hydroxy)phenoxy-3H-xanthene-3-on-9-yl]benzoic acid	[M + H-H_2_O] ^+^	2.80
20	14.10	13.76	13.08	15.32	5.661	6.46	181.0031	5-Chloro-3-methylcatechol	[M + Na]^+^	0.40
								2-Oxopropyl-CoM	[M + H-H_2_O] ^+^	3.80
								Urea phosphate salt	[M + Na]^+^	4.60
21	14.02	13.27	14.63	16.63	5.645	9.05	403.1393	2′,4′,6′-Trihydroxydihydrochalcone 2′-glucoside	[M + H-H_2_O]^+^	0.00
								Rhaponticin	[M + H-H_2_O]^+^	0.00
								Glycyphyllin	[M + H-H_2_O]^+^	0.00
								4,2′-Dihydroxychalcone 4-glucoside	[M + H]^+^	0.60
								7-Hydroxyflavanone beta-D-glucopyranoside	[M + H]^+^	0.60
22	11.41	10.16	12.52	17.13	5.594	8.27	425.0803	5-Demethylmelibentin	[M + Na]^+^	−4.00
								Gossypetin 3,7,3′-trimethyl ether 8-acetate	[M + Na]^+^	−4.00
								5,2′,5′-Trihydroxy-3,7,8-trimethoxyflavone 2′-acetate	[M + Na]^+^	−4.00
								Pelargonidin 3-arabinoside	[M + Na]^+^	−4.00
23	9.95	9.65	13.74	17.65	5.569	8.21	730.1485	p-Coumaroyl vitisin A	[M + Na]^+^	−1.90
24	10.09	10.10	11.21	14.66	5.563	7.09	302.0256	Unknown		
25	9.61	10.26	9.90	15.94	5.557	6.51	239.0457	Bisnorbiotin	[M + Na]^+^	−0.40
								D-erythro-1-(Imidazol-4-yl)glycerol 3-phosphate	[M + H]^+^	3.00
26	9.59	9.00	13.70	17.25	5.535	8.24	687.1237	Isorhamnetin 3-(4′′-sulfatorutinoside)	[M + H-H_2_O]^+^	0.60
27	11.46	11.12	13.15	17.09	5.496	7.82	179.0236	Tetrahydroxypteridine	[M + H-H_2_O]^+^	0.60
								Xanthine-8-carboxylate	[M + H-H_2_O]^+^	0.60
28	8.41	8.80	12.76	16.92	5.460	8.23	286.0291	Unknown		

The data in LIP INI, NO LIP INI, LIP FIN and NO LIP FIN columns refers to mean intensity of metabolites and are presented as log 2

*Abbreviations*: *nd* no detected, *RT* retention time, *VIP* variable importance in projection

**Table 3:** Putative metabolites in ESI-

	NO LIP INI	LIP INI	NO LIP FIN	LIP FIN	VIP	RT (min)	Detected mass (m/z)	Putative metabolites	Assignation	Mass difference (mDa)
1	nd	nd	15.10	19.27	4.552	9.06	385.0878	Phe-Met-OH	[M-H_2_O-H]^−^	2.00
2	nd	nd	16.02	19.71	4.487	9.04	767.1824	Kaempferol 3-(2′′-(E)-feruloylgalactosyl--glucoside)	[M-H_2_O-H]^−^	0.10
								Isoorientin 2′′-(feruloyl-glucoside)	[M-H_2_O-H]^−^	0.10
								Peonidin 3-[6-(3-glucosylcaffeyl)glucoside]	[M-H_2_O-H]^−^	0.10
								Petunidin 3-(6′′-p-coumarylglucoside)-glucoside	[M-H_2_O-H]^−^	0.10
								Isoorientin 4′-O-glucoside 2′′-O-(E)-ferulate	[M-H_2_O-H]^−^	0.10
								Cyanidin 3-(6′′-ferulylglucoside)-glucoside	[M-H_2_O-H]^−^	0.10
3	11.01	10.86	11.99	15.37	4.465	8.29	821.1515	Unknown		
4	14.82	15.42	18.71	22.55	4.348	9.05	383.0907	8-p-Coumaroyl-3,4-dihydro-5,7-dihydroxy-4-phenylcoumarin	[M-H_2_O-H]^−^	−1.20
								Asp-OHPhe-OH	[M-H_2_O-H]^−^	2.80
								Val-His-OH	[M-H_2_O-H]^−^	2.80
5	nd	nd	13.92	17.51	4.127	9.05	769.1913	Unknown		
6	nd	nd	10.30	16.10	4.063	9.05	481.0584	Unknown		

The data in LIP INI, NO LIP INI, LIP FIN and NO LIP FIN columns refers to mean intensity of metabolites and are presented as log 2

*Abbreviations*: *nd* no detected, *RT* retention time, *VIP* variable importance in projection

Correlations between phenotypical parameters and putative metabolites might offer information on whether there is a direct or inverse relationship between them (Table 4). Remarkably interesting is the negative correlation between metabolite 14 and ADMA, FFA and β-hydroxybutyrate, or the positive with SOD, which support the interplay of α-LA administration with the oxidative status.

**Table 4:** Correlation analysis between putative metabolites and biochemical measurements

Putative metabolites	Biochemical measurements	rho	p
Metabolite 14 ESI+	Δ FFA	−0.3621	0.0453
	Endpoint β-hydroxybutyrate	−0.3601	0.0466
	Endpoint ADMA	−0.4397	0.0133
	Endpoint SOD	0.4036	0.0270

rho: Spearman correlation coefficient; p < 0.05 was considered significant; Δ: change: [(endpoint-baseline) / baseline] × 100

*Abbreviations*: *ESI+* positive electrospray ionization, *HOMA-IR* homeostasis model assessment, *FFA* free fatty acids, *ADMA* asymmetric dimethylarginine, *SOD* superoxide dismutase

## Discussion

The higher reductions on BMI and fat mass in those groups supplemented with α-LA could be explained by direct or indirect effects of this organosulfur compound derived from caprylic acid on adipocyte metabolism, regulation mitochondrial biogenesis, lipid turnover (lipolysis/lipogenesis) or inflammation [18, 23] as well as related to its role as antioxidant [37], and its beneficial effects on hyperlipidemia [38], or cardiovascular risk [39].

Metabolomics have been applied for pattern recognition and characteristic metabolite identification [40, 41], as well as for dietary adherence [27] metabolic fingerprinting [42], disease monitoring [43], and post-treatment outcomes [44]. Untargeted metabolomic analyses of urine samples collected at baseline and at the endpoint in a nutritional intervention might offer information on the presence of discriminant metabolites among experimental groups, since metabolomics has contributed to decipher body responses to different treatments in subjects with obesity [45], diabetes [46], fatty liver [47] and COPD [48]. Some of these discriminant metabolites, including amino acids and peptides, lipid species or food derivatives, might be established as biomarkers for subsequent studies [49–51], and have been described in some situations of changes in adiposity [52] or inflammation [53]. In this context, the assessment of the effects of α-LA (derived from a carboxylic acid with 8C) and EPA (20C) administration to obese/overweight women during weight loss [21] may beneficiate from metabolomics approaches.

Discriminant metabolites among the different groups suggest that α-LA has an outstanding importance in the urinary metabolomic profile, independently of the effect of energy restriction. Therefore, the discriminating metabolites among the groups should be mainly related to the intake of α-LA. Although to a lesser extent, the discriminant metabolites could be associated with the greater reduction of body weight, changes in lipid metabolism and insulin sensitivity observed in LIP groups. In any case, the involvement of the slightly, but significant higher weight loss observed in the groups supplemented with α-LA could not be discarded [45] or the role of obesity itself, since amino acids, fatty acids and other species may be involved [54–56].

In this case, the discriminant metabolites are increased in LIP group at endpoint with respect to the other groups, suggesting that protein/lipid catabolism is increased with α-LA supplementation. In this context, previous studies have suggested lipolytic actions of α-LA both in cultured adipocytes [57] and after dietary supplementation [19]. Moreover, α-LA administration has been reported to affect glucose metabolism by inhibiting glycolysis and Thr-Gly-Ser pathways [58] as well as providing carbon groups to the tricarboxylic acid cycle [59]. Furthermore, α-LA (combined with flaxseed oil) appeared to ameliorate hepatic oxidative stress and lipid accumulation [60] or decrease LDL oxidation [61] in addition to its recognized antioxidant properties [37]. Indeed, antioxidants may modulate oxidative stress and inflammatory responses through interrelated mechanisms [62]. Therefore, dietary α-LA may collaborate with endogenous antioxidant machineries on preventive or repair system defenses, where interactions and overlappings with other exogenous antioxidants may occur [63].

The presence of 2,3-diketo-L-gulonate (one isomer of trihydroxy-dioxohexanoate) in urine [64] has been previously described, and has been characterized as an intermediary metabolite of the ascorbic acid metabolism [65, 66]. Plasma levels of α-LA and ascorbic acid have been linked in previous studies [67, 68], revealing that α-LA may improve endogenous ascorbate levels indirectly by inducing its uptake from the blood plasma [67] or by affecting ascorbate recycling [65]. This fact might be associated with antioxidant properties of both compounds, since α-LA acts as antioxidant not only directly through radical quenching and metal chelation, but also indirectly through recycling of other antioxidants such as ascorbate [68] or by increasing the expression of antioxidant enzymes [69]. Furthermore, other isomers such as 2,5-didehydro-D-gluconate (isomers of trihydroxy-dioxohexanoate) or dihydroxy-oxohexanedioate (5-dehydro-4-deoxy-D-glucarate; 2-dehydro-3-deoxy-D-glucarate) with compatible m/z values have been related with ascorbic acid synthesis and degradation [70]. Other metabolites that initially showed similar theoretical mass but discarded after appropriate validation analyses were citrate/isocitrate, succinate and glucaric acid lactone. Therefore, all these findings could explain the presence of an ascorbate intermediate in urine in a supplementary nutritional intervention trial, contributing to overcome and complement the antioxidant properties of ascorbic acid as a reducing agent, which donate electron to various enzymatic and non-enzymatic reaction related to oxidative stress [71]. Interestingly, both α-LA and vitamin C administration have shown reasonable evidence for obesity management [72]. Another issue to be considered is that the structure of ascorbic acid (C_6_H_8_O_6_) is close to glucose/monosaccharides, while α-LA has regulatory functions on the Krebs cycle [37, 73].

Correlations between metabolite 14 and ADMA or SOD could be mediated by the antioxidant power of α-LA, previously described in several studies [68], and also involving benefits on body adiposity [18, 21], where interactions with vitamin C cannot be discarded [74]. Finally, correlations metabolite 14 with FFA and hydroxybutyrate [75] could be associated with less BMI observed in α-LA supplementation groups at endpoint.

## Conclusions

Summing up, this metabolomic approach supports the hypothesis that the beneficial effects of α-LA administration on body weight reduction may be partly explained by the antioxidant properties of this organosulfur carboxylic acid, where interactions with ascorbic acid should be taken into account mediated by trihydroxy-dioxohexanoate or dihydroxy-oxohexanedioate.

## Additional file

Additional file 1 : **Figure S1.** Principal component analysis (PCA) of untargeted metabolomics analysis of urine samples, including CONTROL (red), EPA (green), LA (dark blue), EPA+LA (light blue) groups. A) PCA in positive ionization mode (ESI+). B) PCA in negative ionization mode (ESI-). (DOCX 199 kb)

## Abbreviations

α-LA: α-lipoic acid
ADMA: asymmetric dimethylarginine
BMI: body mass index
EPA: eicosapentaenoic acid
ESI: electrospray ionization
FFA: free fatty acids
HPLC-TOF-MS: liquid chromatography coupled with time of flight mass spectrometry
incremental area under the curve HOMA-IR: homeostasis model assessment
LIP: lipoic group
MetS: metabolic syndrome
NO LIP: no lipoic group
PCA: principal component analysis
PLS-DA: partial least squares-discriminant analysis
SOD: superoxide dismutase
VIP: variable importance in projection
