# Supplementary material for: Untargeted metabolomic on urine samples after α-lipoic acid and/or eicosapentaenoic acid supplementation in healthy overweight/obese women
Source: Lipids Health Dis. 2018 May 9;17:103. doi: 10.1186/s12944-018-0750-4 (PMC5941619; doi:10.1186/s12944-018-0750-4)
Supplement: Supplementary file 1 — : Figure S1. Principal component analysis (PCA) of untargeted metabolomics analysis of urine samples, including CONTROL (red), EPA (green), LA (dark blue), EPA+LA (light blue) groups. A) PCA in positive ionization mode (ESI+). B) PCA in negative ionization mode (ESI-). (DOCX 199 kb) [file 12944_2018_750_MOESM1_ESM.docx]

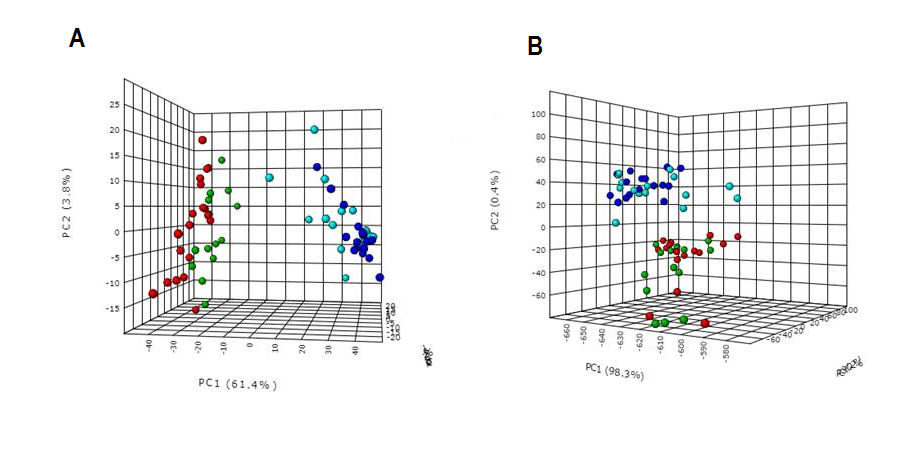


**Additional file 1: Figure S1.** Principal component analysis (PCA) of untargeted metabolomics analysis of urine samples, including CONTROL (red), EPA (green), LA (dark blue), EPA+LA (light blue) groups. A) PCA in positive ionization mode (ESI+). B) PCA in negative ionization mode (ESI-).
